# Unripe Plantain Peel Biohydrogel for Methylene Blue Removal from Aqueous Solution

**DOI:** 10.3390/polym16223135

**Published:** 2024-11-10

**Authors:** Andrés Felipe Chamorro, Sixta Palencia Luna, Manuel Palencia

**Affiliations:** 1 Research Group of Electrochemistry and Environment (GIEMA), Faculty of Basic Sciences, Universidad Santiago de Cali, Cali 760035, Colombia; 2 Department of Biological and Chemical Sciences, Faculty of Medicine and Biological Sciences, Universidad San Sebastián, Concepción 4030000, Chile; sixta.palencia@uss.cl; 3 Mindtech Research Group (Mindtech-RG), Mindtech s.a.s, Cali 25360, Colombia; 4 Research Group in Science with Technological Applications (GICAT), Department of Chemistry, Faculty of Natural and Exact Science, Universidad del Valle, Cali 25360, Colombia

**Keywords:** hydrogels, biohydrogels, methylene blue, dyes and pigments, dye removal, adsorption, banana peel, biosorption

## Abstract

Dye contamination is a serious environmental issue, particularly affecting water bodies, driving efforts to synthesize adsorbent materials with high dye-removal capacities. In this context, eco-friendly and cost-effective materials derived from bioresidues are being explored to recycle and valorize waste. This study investigates the synthesis, characterization, and application of a biohydrogel made from unripe plantain peel (PP), modified with carboxymethyl groups and crosslinked using varying concentrations of citric acid (CA), an eco-friendly and economical organic acid. The materials were characterized by ATR-FTIR, TGA, and SEM, confirming the successful synthesis of hydrogels, which exhibited rough, irregular surfaces with micropores. Additionally, the materials were analyzed for their pH point of zero charge, swelling capacity, and methylene blue (MB) dye removal efficiency. The results indicate that the biohydrogel formed with 1% CA exhibited the most favorable characteristics for MB removal. Kinetic studies revealed that the adsorption mechanism is pH-dependent, with equilibrium being reached in 720 min. The Freundlich isotherm model provided the best fit for the adsorption data, suggesting a heterogeneous surface and a multilayer adsorption process, with a maximum retention capacity of 600.8 ± 2.1 mg/g at pH 4. These findings contribute to the development of cost-effective and efficient materials for dye removal, particularly from water bodies.

## 1. Introduction

Dyes and pigments are widely used across various industries globally, including textiles, paint, food, and paper production, among others [1,2]. Currently, more than 1000 dyes are in use worldwide, with large quantities accumulating in water bodies [3,4]. Dyes negatively impact aquatic environments because they are persistent and highly resistant to thermal and light degradation [5]. For instance, dyes can form films on the surface of water bodies, preventing sunlight from penetrating the water, which disrupts the photosynthetic processes of aquatic flora and fauna [6,7]. Furthermore, many dyes containing aromatic, carboxyl, and sulfonated groups are highly toxic to humans, causing skin irritation, cardiovascular diseases, and even mutations [8]. For example, MB, commonly used in the textile industry to dye cotton and silk, can cause permanent damage to the eyes of both humans and animals. It can also lead to breathing difficulties, vomiting, nausea, and mental confusion [9]. As a result, developing effective methods for dye removal from water bodies has become a significant challenge.

Several physicochemical methods have been developed to remove dyes from water bodies, including physical processes such as ultrafiltration, ozonation, and membrane separation [10,11]; biological treatments using microbes, fungi, enzymes, and bacteria [12]; and photocatalysts to reduce the toxicity of dyes [13]. However, many of these methods are expensive, making large-scale applications challenging [9]. In contrast, dye removal through adsorption has emerged as an efficient and economical alternative. Hydrogels have demonstrated high dye removal efficiency and ease of reuse [1]. These cross-linked polymeric materials form three-dimensional (3D) networks, exhibit high elasticity, and have the ability to swell in hydrophilic solvents [14]. Synthetic polymeric hydrogels have been explored for dye retention; for example, polyvinyl alcohol-glutaraldehyde cross-linked hydrogel beads have been studied for the retention of Congo Red dye [7]. However, these materials show limited retention capacity for dyes, with a maximum of 34 mg/g at pH 6. Additionally, the high cost and limited biodegradability of synthetic polymers hinder their widespread application.

In contrast, hydrogels formed from biopolymers are emerging as a superior alternative for dye removal, as biopolymers are biodegradable, non-toxic, and environmentally compatible, reducing collateral toxicity in water bodies. For example, an alginate hydrogel with montmorillonite clay beads demonstrated an MB removal capacity of 678.2 mg/g at pH 7 and 30 °C [15]. Another example is the use of carboxymethyl cellulose nanocrystals to remove MB from water samples, achieving a high removal capacity of 756 mg/g at pH 11 [16]. However, obtaining biopolymers such as cellulose, alginate, and chitosan typically requires chemical treatment of raw materials, which increases the cost of applying these materials for dye adsorption. Therefore, a promising alternative is the use of raw biowaste without prior extraction to obtain biopolymers, such as bioresidues from plantain consumption. Thus, plantains can be chemically modified to promote interaction with pollutants.

Plantain production amounts to around 125 million tons per year globally [17], with approximately 40% of the fresh fruit weight consisting of PP, which becomes a solid waste rich in biopolymers such as cellulose, hemicellulose, and lignin [18]. This raw material, without prior treatment, can be chemically modified for use in dye removal from water bodies. To develop an economical, biodegradable, and environmentally compatible material for dye removal, this study aimed to produce carboxymethylated PP biowaste crosslinked with CA (CPPCA) and evaluate its capacity to remove cationic dyes. MB was used as a model dye to assess removal efficiency across different pH levels. Additionally, the MB adsorption data were analyzed using adsorption and kinetic models to better understand the adsorption mechanism.

## 2. Materials and Methods

### 2.1. Materials

Unripe plantain peel was sourced from a local market in Cali, Colombia. Chloroacetic acid (C_2_H_3_ClO_2_), sodium hydroxide (NaOH), acetic acid (CH_3_COOH), citric acid (C_6_H_8_O_7_), methanol (CH_3_OH), ethanol (C_2_H_6_O), and methylene blue (C_16_H_18_ClN_3_S) were purchased from Sigma-Aldrich (St. Louis, MO, USA).

### 2.2. CPPCA Hydrogels Formation and Cellulose Obtention from Banana Peel (CSPP)

The PP was dried for 20 days under sunlight, then milled and sieved to a particle size of 2 mm. To obtain CSPP, PP was treated with 15% NaOH for 2 h under mechanical stirring, followed by bleaching with 15% NaClO. The material was then immersed in 1% H_2_SO_4_ under stirring for 1 h. After treatment, the resulting material was washed with distilled water and dried at 45 °C for 92 h. On the other hand, to form the carboxymethylated PP (CPP), 10 g of PP was mixed and stirred with 8.1 g of sodium hydroxide (NaOH) and 75 mL of ethanol for 1 h. Next, 24.3 g of chloroacetic acid was added, and the reaction was continuously stirred at 55 °C under reflux for 2 h. The CPP was filtered and washed with 200 mL of methanol. The resulting product was dispersed in water and neutralized with acetic acid. The CPP was filtered again and washed thoroughly with ethanol. Finally, the material was dried for 48 h at 60 °C. To form the CPPCA, we followed the methodology used by [19]. A total of 10 g of CPP was dispersed in 25 g of distilled water and heated to 60 °C. A predetermined quantity of CA was then added to achieve a CA concentration, and the mixture was heated at 60 °C for 1 h. The resulting material was filtered, washed with distilled water, and then dried at 60 °C for 72 h. This process was repeated with varying CA concentrations, yielding CPPCA(x), where x refers to CA concentrations ranging from 1% to 10% *w*/*w*.

### 2.3. Physicochemical and Morphological Characterization of Hydrogel

Fourier transform infrared spectroscopy (FTIR) with attenuated total reflectance (ATR) was employed to characterize cellulose from PP, as well as CPP and CPPCA(x) hydrogels (IR-Affinity-1, Shimadzu, Kyoto, Japan). The spectra were collected to confirm the etherification and esterification reactions, with data acquisition spanning from 4000 to 700 cm^−1^ at 2 cm^−1^ intervals, and 20 scans were conducted per sample. Thermal properties were examined using thermogravimetric analysis (TGA, SDT-Q600, TA Instruments, New Castle, DE, USA) for cellulose from PP, CPP, and CPPCA(1). Approximately 5–10 mg of each material was heated from ambient temperature to 700 °C at a rate of 10 °C/min under a nitrogen flow of 20 mL/min. The analysis focused on the first-order derivative of the raw thermogram to interpret the weight loss results. Finally, the morphology of the materials was analyzed using digital photography with a flexible digital electronic microscope (Suzhou Jingtong Instrument Co., Ltd., Suzhou, China) and scanning electron microscopy (SEM, Thermo Fisher Scientific, Phenom Pro X, Waltham, MA, USA).

#### 2.3.1. Determination of the Water Absorption Capacity (WAC)

WAC was measured using the tea bag technique [20]. A pre-weighed tea bag was filled with a specific amount of CPPCA(x) and submerged in water for 24 h. After immersion, the tea bag containing the hydrogel was suspended for 15 min to allow excess water to drain by gravity. The final weight was recorded, and WAC was calculated using Equation (1). A control experiment was also conducted using an empty tea bag.

(1) $$WAC=\frac{w_{2}-(w_{1}+w_{0})}{w_{1}}$$

In this method, w_0_, w_1_, and w_2_ represent the masses of the empty tea bag, the CPPCA(x) sample, and the tea bag containing the CPPCA(x) after water absorption, respectively. Each experiment was conducted in triplicate for accuracy.

#### 2.3.2. Determination of the Gel Fraction

The gel fraction was measured following the method described by [21]. To determine the gel fraction, a pre-weighed sample of CPPCA(x) was placed in a beaker and submerged in distilled water for 24 h. After this period, the water was drained, and the sample was dried in an oven at 50 °C until a constant weight was achieved. The gel fraction was then calculated using Equation (2).

(2) $$Gel\;fraction=\frac{w_{f}}{w_{0}}\times 100$$

where W_f_ and W_0_ represent the final dried weight and the initial dried weight, respectively. This value indicates the effectiveness of the CA crosslinking process.

#### 2.3.3. Determination of the Point of Zero Charge (pH_PZC_) of the CPPCA(1)

The pH_PZC_ for the CPPCA(1) was determined using the methodology described by [22,23]. A 0.2 g sample of CPPCA(1) was immersed in a 0.100 mol/L NaCl solution, previously adjusted to different pH levels (2.0, 4.0, 6.0, 8.0, 10.0, and 12.0). The mixtures were stirred at 25 °C for 24 h, after which the pH of the resulting supernatant was measured. The difference between the initial and final pH (ΔpH) was plotted against the initial pH to determine the pH_PZC_. All experiments were performed in duplicate.

### 2.4. Dye Adsorption Studies

#### 2.4.1. Dye Removal Efficiency

To assess the adsorption capacity, approximately 0.2 g of CPP and CPPCA(x) were introduced into 10.0 mL of a dye solution (100 mg/L) and agitated in a water bath at 100 rpm for 24 h to reach equilibrium. The dye concentration in the supernatant was then determined using an ultraviolet–visible spectrophotometer (600 Plus, Merck, Darmstadt, Germany). A linear calibration curve, ranging from 0.5 to 5.0 mg/L (see Figure S1), was used for quantification at 665 nm, corresponding to the absorbance maximum as reported in the literature [24,25]. The removal efficiency percentage (% RE) was calculated using Equation (3).

(3) $$\%RE=\frac{C_{1}-C_{2}}{C_{1}}\times 100$$

In this equation, C_1_ and C_2_ represent the concentrations of the dye solution (mg/L) before and after the adsorption process, respectively [26]. The % RE for each material was calculated in triplicate to ensure accuracy.

#### 2.4.2. Adsorption Kinetics

The effect of contact time on dye adsorption was studied over a period ranging from 30 to 2880 min, allowing the calculation of adsorption capacity at each time point (q_t_) using Equation (4):

(4) $$q_{t}=\frac{\left(C_{0}-C_{t}\right)V}{m}$$

where C_0_ and C_t_ (mg/L) represent the initial dye concentration and the concentration at time t, respectively. V (L) is the volume of the dye solution, and m (g) is the mass of the adsorbent. To analyze the adsorption kinetics, the experimental data were modeled using the pseudo-first-order, pseudo-second-order, Boyd liquid-film diffusion, and Elovich models, as described by Equations (5)–(8).

(5) $$\log\left(q_{e}-q_{t}\right)=\log q_{e}-\frac{k_{1}}{2.303}t$$

(6) $$\frac{t}{q_{t}}=\frac{1}{k_{2}q_{e}^{2}}+\frac{t}{q_{e}}$$

(7) $$-\ln\left(1-\frac{q_{t}}{q_{e}}\right)=k_{FD}t$$

(8) $$q_{t}=\frac{1}{\beta }\ln(\alpha \beta )+\frac{1}{\beta }\ln t$$

In these equations, q_e_ (mg/g) and q_t_ (mg/g) represent the adsorption capacities at equilibrium and at time t (min), respectively. The constants k_1_ (1/min) and k_2_ (g/(mg min)) are the pseudo-first-order and pseudo-second-order rate constants, respectively, while k_FD_ (1/min) represents the Boyd liquid-film diffusion rate constant. In the Elovich model, β and α correspond to the desorption constant (g/mg) and the initial adsorption rate (mg/g·min), respectively [27].

#### 2.4.3. Adsorption Capacity

The adsorption process was studied following the methodology outlined in Section 2.4.1 at 25 °C, with varying pH levels (2, 4, 7, and 10). The equilibrium adsorption capacity (q_e_, mg/g) for different initial dye concentrations (0–20,000 mg/L) was calculated using Equation (9).

(9) $$q_{e}=\frac{\left(C_{o}-C_{e}\right)V}{m}$$

where C_o_ (mg/L) and C_e_ (mg/L) represent the initial and equilibrium dye concentrations, respectively. V (L) refers to the volume of the dye solution, and m (g) is the mass of the dried CPPCA(1) hydrogel. To better understand the interaction mechanism between the dye and the hydrogel, the Langmuir, Freundlich, Temkin, and Redlich–Peterson adsorption isotherms were evaluated using Equations (10)–(13).

(10) $$\frac{C_{e}}{q_{e}}=\frac{1}{\left(q_{m}K_{L}\right)}+\frac{C_{e}}{q_{m}}$$

(11) $$\ln q_{e}=\ln K_{F}+\frac{1}{n}\ln C_{e}$$

(12) $$q_{e}=B_{T}\ln K_{T}+B_{T}\ln C_{e}$$

(13) $$\ln\left(\frac{C_{e}}{q_{e}}\right)=B\ln C_{e}-\ln K_{R-P}$$

In these equations, q_m_ and q_e_ (mg/g) represent the maximum adsorption capacity and the adsorption capacity at equilibrium, respectively, while C_e_ (mg/L) is the equilibrium dye concentration. For the Langmuir model, K_L_ (L/mg) is the Langmuir constant, which is related to adsorption energy and affinity. The Freundlich constants K_F_ (L/mg) and n describe the adsorption capacity and intensity, respectively. In the Temkin model, K_T_ is the binding constant, and B is the Temkin constant, which depends on temperature (J mol^−1^). Lastly, K_R-P_ is the Redlich–Peterson constant (L/g), and B (L/mg) is the Redlich–Peterson parameter, which describes adsorption capacity and the interaction between adsorbate and adsorbent, respectively [28].

#### 2.4.4. Adsorption Thermodynamic Parameters

The adsorption thermodynamic parameters were determined for methylene blue (MB) on the CPPCA(1) hydrogel by evaluating the adsorption at different temperatures (25–50 °C) following the methodology reported in Section 2.4.1, with an MB concentration of 2000 mg/L. The standard Gibbs free energy change (∆G°), the standard enthalpy change (∆H°), and the standard entropy change (ΔS°) were calculated using the Equations (14)–(16) [29,30]:

(14) $$K_{c}=\frac{q_{e}}{C_{e}}$$

(15) $$∆G^{{}^{\circ}}=-RT\ln K_{c}$$

(16) $$\ln K_{c}=\frac{∆S^{{}^{\circ}}}{R}-\frac{∆H^{{}^{\circ}}}{RT}$$

where K_c_, R, and T represent the distribution coefficient at different temperatures, the gas constant (8.314 J/mol K), and the temperature (K), respectively. The thermodynamic parameters, $∆H^{{}^{\circ}}$ and $∆S^{{}^{\circ}}$, can be obtained from the slope and intercept of the linear plot of $\ln K_{c}$ versus 1/T.

#### 2.4.5. Adsorption Tracking

To develop a model for adsorption behavior at different pH values, a multilevel factorial experimental design and response surface methodology were utilized to analyze how the variable q_e_ is influenced by initial concentration (Ci) and pH. The relationship was represented using a second-order polynomial Equation (17).

(17) $$Y=\beta_{o}+\sum_{i=1}^{k}\beta_{i}x_{i}+\sum_{i=1}^{k}\beta_{ii}x_{i}^{2}+\sum_{i=1}^{k-1}\sum_{j=2}^{k}\beta_{ij}x_{i}x_{j}+\varepsilon$$

In this equation, x_i_, x_j_, …, x_k_ denote the factors affecting the response variable Y, while the terms x_i_^2^, x_j_^2^, and x_k_^2^ indicate the quadratic effects. The terms x_i_ x_j_, x_i_ x_k_, and x_j_ x_k_ reflect the interaction effects. The parameters β_0_, β_i_, β_ii_, β_ij_, and ε correspond to the intercept term, linear effect coefficients, squared effect coefficients, interaction coefficients, and random error, respectively [31,32].

## 3. Results and Discussion

### 3.1. CPPCA Hydrogel Formation and Characterization

**Figure 1:**
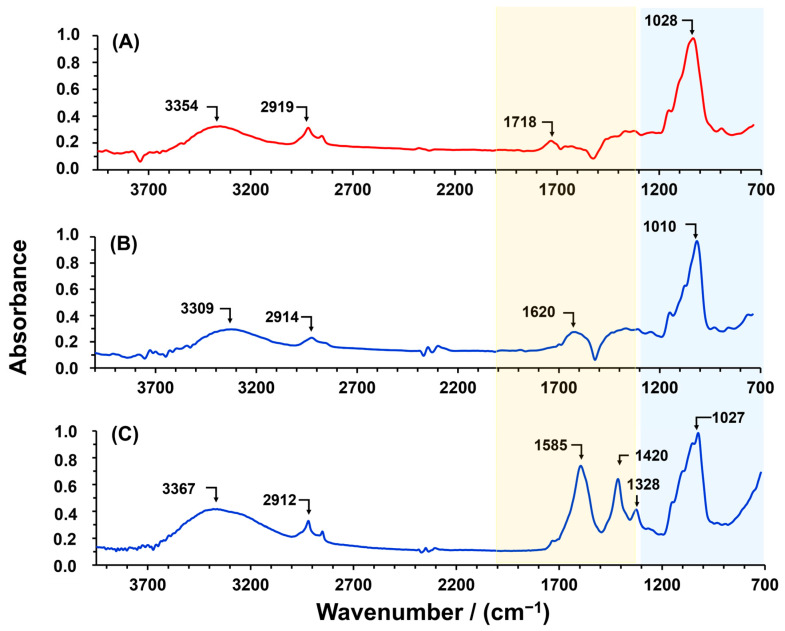
ATR-FTIR spectra of (**A**) CSPP, (**B**) PP, and (**C**) CPP.

Figure 1 shows the ATR-FTIR spectra of CSPP (cellulose obtained from banana peel), PP, and CPP. In the spectra of CSPP (Figure 1A), characteristic bands of polysaccharides appear, highlighting the bands at 3354 cm^−1^ and 2919 cm^−1^, which are attributed to the O-H and C-H stretching vibrations of the glucose units, respectively. Moreover, at 1010 cm^−1^ the symmetric and asymmetric stretching of the -C-O-C- in the pyranose ring of cellulose obtained from PP is observed. Additionally, at 1718 cm^−1^, there is a band possibly attributed to the symmetric stretching of carboxylic groups (C=O) from hemicellulose residues in the CSPP. On the other hand, in the ATR-FTIR spectrum of PP (Figure 1B), a distinct band at 1620 cm^−1^ is observed, which is assigned to the stretching vibrations of carbonyl groups present in the acid and aldehyde functional groups of lignin. Comparing the CSPP and PP spectra reveals similar profiles. Therefore, to reduce costs in obtaining carboxymethyl cellulose for the removal of pollutants, PP could be utilized as a raw material to produce an economical material rich in carboxymethyl groups with a higher affinity for cationic pollutants.

**Figure 2:**
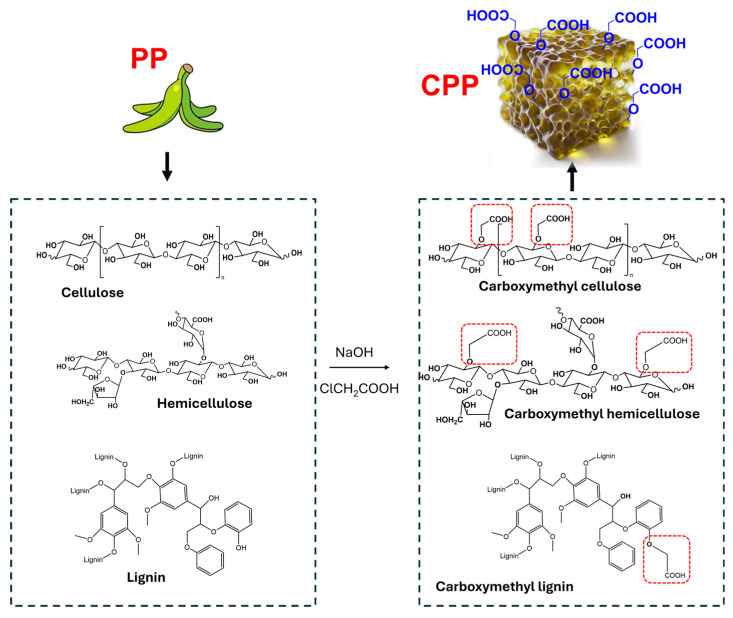
Schematic representation of the carboxymethylation reaction of cellulose, hemicellulose, and lignin from PP to obtain the hydrogel CPP.

Figure 1C shows the ATR-FTIR spectra of CPP. The intense band at 1585 cm^−1^ is assigned to the symmetric stretching of the carbonyl bond (C=O) of the carboxymethyl substituent, which appears at a lower wavenumber due to the presence of the electronic pair on the adjacent oxygen [33]. Additionally, the previously mentioned bands, along with the band at 1420 cm^−1^, which is assigned to carboxylate stretching vibrations, indicate the successful chemical modification of the PP [34]. Furthermore, the band at 1328 cm^−1^ is attributed to the vibration of -O-H [35]. In comparison to PP, the band assigned to the -C-O-C- stretching vibration in the pyranose ring shifted to 1027 cm^−1^, likely due to the effect of the electron pair of the oxygen in the carboxymethyl groups. However, the intense band indicates that the carbohydrates in the PP (cellulose and hemicellulose) were not degraded during the chemical modification. Although this research did not characterize the chemical composition of the PP, the literature reports on the characterization of different banana species indicate that, on average, PP is composed of carbohydrates such as cellulose (~19%), hemicellulose (~20%), and lignin (~17%) [36,37,38,39]. Consequently, carboxymethyl groups could modify the hydroxyl groups of the aforementioned biopolymers (Figure 2). For instance, carboxymethyl lignin and carboxymethyl hemicellulose have been synthesized using the same methodology employed in this research [40,41,42], indicating that the modification of PP could lead to the formation of these biopolymers in addition to carboxymethyl cellulose.

**Figure 3:**
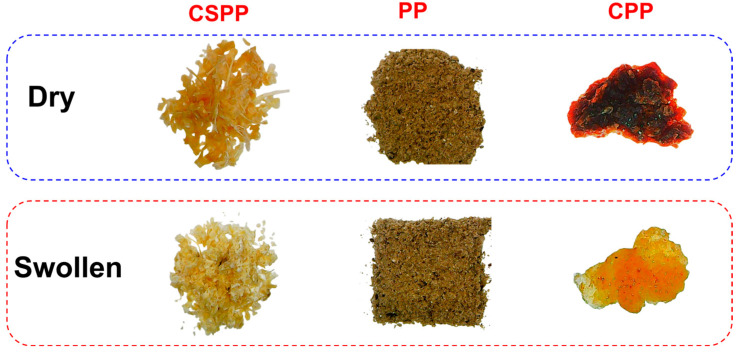
Digital photography of CSPP, PP, and CPP dry and swollen after 24 h in distillated water.

Figure 3 presents photographs of the CSPP, PP, and CPP before and after immersion in distilled water for 24 h. It can be observed that both CSPP and PP do not retain water; however, the CPP shows an increase in size and water retention, indicating the formation of a gel structure. Notably, when the CPP is dispersed in water under magnetic agitation (see Figure S2), it affects the material’s application for pollutant retention. To address this issue and obtain a non-dispersive material, the CPP was crosslinked with CA, a non-toxic acid. Furthermore, CA is a green and economical reactant [43], enabling its use without significantly increasing the production costs of the material.

**Figure 4:**
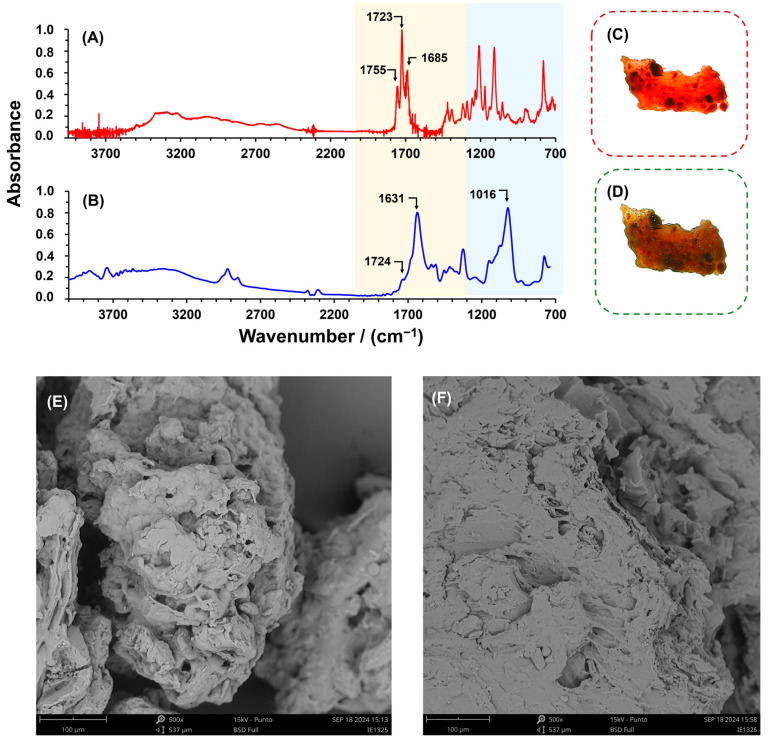
ATR-FTIR spectra of (**A**) CA and (**B**) CPPCA(1). Digital photographs of (**C**) the dry form and (**D**) the swollen form of CPPCA(1) after 24 h of immersion in distilled water. Additionally, SEM images are presented for (**E**) CPP and (**F**) CPPCA(1).

Figure 4A,B display the ATR-FTIR spectra of CA and CPPCA(1). The CA spectrum reveals three principal bands within the range of 1800 to 1600 cm^−1^, corresponding to the symmetric stretching of the three carboxylic groups in CA, specifically at 1755 cm^−1^, 1723 cm^−1^, and 1685 cm^−1^. In contrast, the CPPCA(1) spectrum shows a representative band at 1016 cm^−1^, which is assigned to the -O-C-O- bonds of the glucose units present in the polysaccharides of the PP. Additionally, the band at 1631 cm^−1^ is attributed to the carbonyl vibration of the carboxymethyl group, while the band at 1724 cm^−1^ corresponds to the symmetric stretching of esters, confirming the polycondensation reaction between the hydroxyl groups of the carboxylic acid groups of CA, which results in the formation of a hydrogel structure. This was further corroborated by immersing the CPPCA(1) in distilled water for 24 h (Figure 4C,D), where it was observed to absorb water and display a rigid structure, significantly decreasing its solubility compared to the CPP, thus enhancing its suitability for dye removal from water. SEM images were taken to observe the morphological differences between the CPP and CPPCA(1) hydrogels (Figure 4E,F). The CPP exhibits a rough and irregular surface with discontinuous microporosity. In contrast, the CPPCA(1) displays a smoother and much denser structure compared to the CPP, attributed to the matrix esterification with CA. Additionally, energy-dispersive X-ray spectroscopy (EDS) results support the crosslinking reaction, as the oxygen proportion in CPPCA(1) (52.7%) is higher than in CPP (33.2%), reflecting the presence of CA in the hydrogel (see Figure S3).

**Figure 5:**
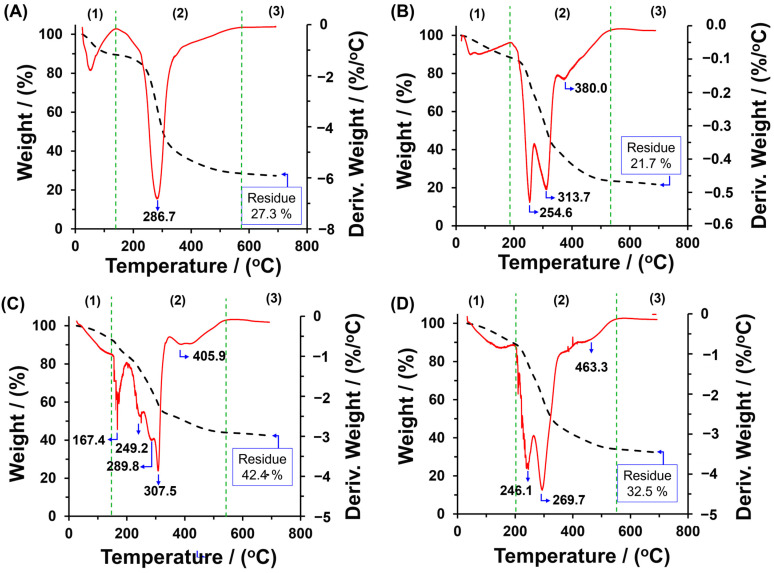
TGA analysis of (**A**) CSPP, (**B**) PP, (**C**) CPP, and (**D**) CPPCA(1).

The materials were thermally characterized using TGA, as shown in Figure 5. All TGA curves exhibited three distinct regions: the first two correspond to degradation, while the third indicates the residual material. Notably, the initial weight loss for all materials occurred from room temperature to approximately 200 °C, reflecting water loss, with an approximate 10% weight reduction observed across all samples. The presence of water in these materials suggests their hydrophilic characteristics, highlighting their capacity to retain water and indicating favorable interactions with hydrophilic pollutants.

In the second degradation region, notable changes in the TGA profiles of the materials were observed. For CSPP (Figure 5A), this degradation region spans approximately 200 to 600 °C, with a maximum degradation temperature of 286.7 °C, resulting in a weight loss of about 62.7%. This stage corresponds to the oxidative thermal degradation of cellulose, leading to the destruction of its structure and a residue of 27.3%. In contrast, the PP exhibited a different profile in this degradation region (Figure 5B), displaying three decomposition temperature peaks at 254.6 °C, 313.7 °C, and 380 °C, which are attributed to the thermal degradation of hemicellulose, cellulose, and lignin, respectively. These profiles align with the TGA results reported by [44], which also identified overlapping peaks in the degradation of PP. The CPP demonstrated a different behavior compared to PP, with several degradation temperatures observed below 300 °C, indicating the loss of carboxylate groups on the polysaccharide (Figure 5C). This weight loss is consistent with findings by Rani et al. (2014) regarding the modification of cellulose from kenaf bast fiber with carboxymethyl groups [45]. The multiple peaks observed in this study suggest the degradation of these functional groups, reflecting modifications across the different polysaccharides present in PP, including hemicellulose, cellulose, and lignin. The substitution of hydroxyl groups with carboxymethyl groups modifies the hydrogen bonding within the crystalline structures of the polysaccharides, weakening the bonding energy and consequently reducing thermal stability [46]. As a result, CPP can be utilized at moderate temperatures below 160 °C, beyond which thermal degradation of the carboxymethyl functional groups occurs. Additionally, the thermogram reveals that the peaks corresponding to cellulose and lignin appear at different temperatures compared to the PP sample. Notably, the CPPCA(1) exhibits thermogram behavior similar to that of PP (Figure 5D), indicating increased thermal stability compared to the CPP material. This enhancement in thermal stability is attributed to the presence of the CA crosslinker in the lignocellulosic matrix, which raises the temperature required to break the crosslinker bonds [47].

To evaluate the effect of CA concentration on the hydrogel, CPPCA hydrogels were formed with varying concentrations of CA ranging from 1% to 10%. The ATR-FTIR spectra confirmed the successful esterification of the lignocellulosic material across all tested CA concentrations (Figure S4). As the CA concentration increased, the band corresponding to the ester bond became more intense, indicating a higher degree of crosslinking. Furthermore, all materials formed with CA exhibited the principal bands associated with carbohydrates, suggesting that there was no degradation of the material during the chemical modification. The esterification of the CPPCA(x) was further corroborated by digital photographs of both the dry and swollen hydrogels. The dry hydrogels displayed a red-brown hue, while the swollen hydrogels increased in size. However, at higher CA concentrations, the material did not exhibit a swollen appearance (Figure S5).

**Figure 6:**
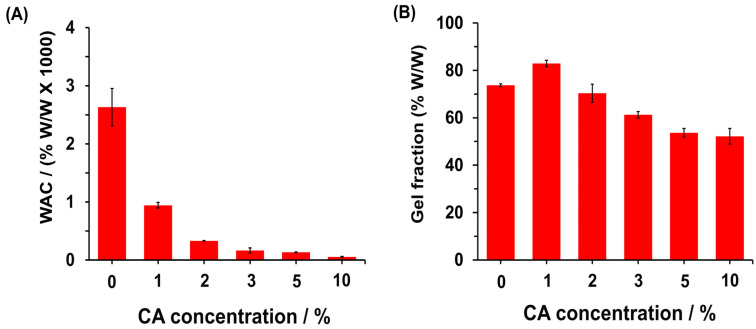
(**A**) WAC and (**B**) gel fraction of CPPCA hydrogels formed at different CA concentrations, ranging from 0 to 10%. The hydrogel formed with 0% of CA corresponds to the CPP.

To quantify the water content in the hydrogels after immersion in water, the WAC was calculated (Figure 6A). The results indicated that CPP exhibited the highest WAC value at 2630.9 ± 321.4%. However, when the material was crosslinked with CA, the WAC decreased significantly, dropping from 943.3 ± 49.5% to 54.7 ± 10.1% as the CA concentration increased from 1% to 10%, respectively. This reduction in WAC suggests that the increased crosslinking degree due to CA limits the hydrogel network’s ability to retain water. Additionally, it was observed that as the CA concentration increased, the solubility of the material decreased after stirring in distilled water for 24 h (Figure S6). Consequently, the gel fraction of the formed material was quantified. For CPP, the gel fraction was 73.7 ± 0.6%, which increased to 82.8 ± 1.4% for CPPCA(1). However, as the CA concentration continued to rise, the gel fraction decreased to 52.2 ± 3.3% for CPPCA(10), indicating that the hydrogels lost unreacted CA and CPP upon immersion in water. Thus, higher CA concentrations induce CPP solubilization. Similar results were observed by our research group in the crosslinking of starch with CA, where the gel fraction dropped to less than 40% at higher CA concentrations [21].

### 3.2. CPPCA Applied to MB Retention

**Figure 7:**
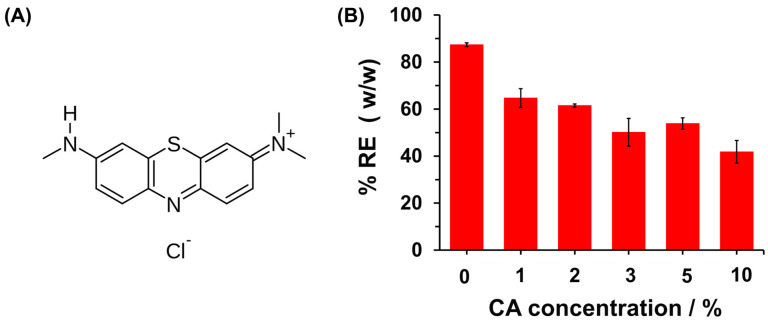
(**A**) Structure of MB and (**B**) % RE of MB on CPPCA hydrogels formed at different CA concentrations, ranging from 0 to 10%. The hydrogel formed with 0% of CA corresponds to the CPP. Experimental conditions: temperature = 25 °C, contact time = 24 h, Co = 100 mg/L, and adsorbent dose = 0.2 g/mL.

To evaluate the hydrogels’ effectiveness in retaining cationic dyes, MB was used as a model cationic dye (Figure 7A). Figure 7B illustrates the dye retention capacity of MB in both CPP and CPPCA hydrogels formed with varying concentrations of CA. The CPP material demonstrated the highest dye retention at 81.1 ± 1.3%, indicating a favorable interaction between the carboxymethyl groups and the cationic dye through electrostatic interactions. However, the dye retention capacity decreased with increasing CA concentration in the hydrogel formation, dropping from 62.2 ± 3.7% for CPPCA(1) to 40.6 ± 4.8% for CPPCA(10). This reduction is likely due to the increased crosslinking degree, which limits the diffusion of the dye into the hydrogel network, thereby decreasing the interactions between the carboxymethyl groups and MB. Although CPP exhibited the highest removal efficiency (% RE), it had a lower gel fraction compared to CPPCA(1), affecting the material’s recovery (Figure 6B). This factor is crucial for scaling the material for industrial applications, especially for its reusability. Therefore, considering the previous results alongside the WAC and gel fraction data, CPPCA(1) emerges as a promising material for the removal of cationic dyes like MB, primarily due to its high dye retention, moderate WAC, and high gel fraction compared to CPP.

#### 3.2.1. Adsorption Kinetics

**Figure 8:**
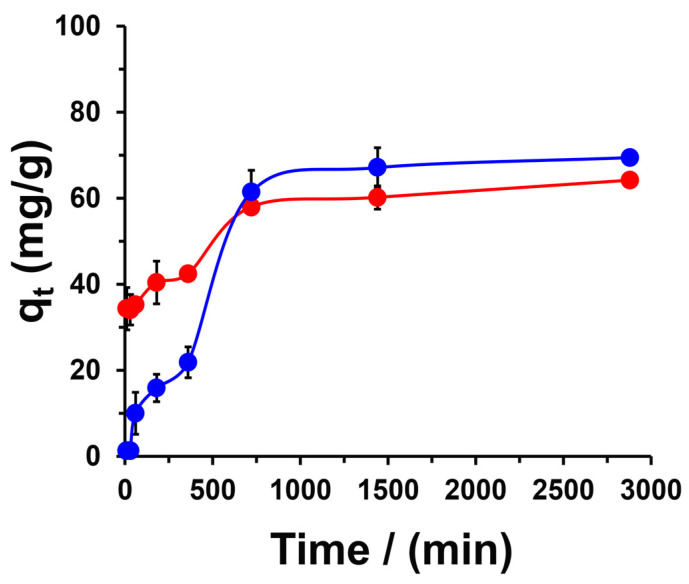
Adsorption kinetics of MB on CPPCA(1) at pH 4 (●) and 7 (●). Conditions: temperature = 25 °C, Co = 2000 mg/L, and adsorbent dose = 0.2 g/mL. The solid lines serve as a visual guide only.

To evaluate the chemical interaction between methylene blue (MB) and CPPCA(1), the adsorption process was analyzed over different time intervals, as adsorption kinetics is a critical factor in assessing the application of the adsorbent material. Prior to the adsorption kinetics assays, the pH_PZC_ of the CPPCA(1) was determined to be 3.6 (Figure S7). This value indicates the pH at which the net charge of the hydrogel surface is zero [23]. Consequently, at pH levels up to 3.6, the hydrogel exhibits a negatively charged surface, while below this pH, the surface carries a positive charge [22]. Considering the favorable electrostatic interaction between the cationic MB and the anionic CPPCA(1), the adsorption kinetics experiments were conducted at pH levels of 4 and 7 (Figure 8).

At pH 7, the dye adsorbs rapidly onto the biohydrogel within the first 10 min of contact, achieving a capacity of q_10min_ = 34.3 ± 3.9 mg/g. A similar trend was observed at pH 4; however, in the initial minutes of contact, the material demonstrated a lower retention capacity compared to pH 7 (q_10min_ < 2 mg/g). Furthermore, both pH curves reached a steady state at approximately 720 min, with retention capacities of 64.2 ± 1.5 mg/g and 69.4 ± 1.2 mg/g for pH 7 and 4, respectively. These results indicate that pH affects the adsorption profile and retention capacity of the CPPCA(1) hydrogel. The adsorption kinetics involved several transport processes, which can be outlined as follows: step (i) transport of the adsorbate to the periphery of the material; step (ii) film diffusion of the adsorbate from the liquid phase to the solid phase (adsorbent material); step (iii) intraparticle diffusion of the adsorbate into the pores and spaces of the material; and step (iv) chemical interaction between the adsorbent and adsorbate, leading to fixation on the material [48]. To better understand the kinetic behavior, various adsorption models were applied to the kinetic data. The kinetic parameters for each model are presented in Table 1. Based on the coefficients of determination (R^2^), the pseudo-second-order and Boyd liquid-film diffusion models exhibited the best fit for pH 7 (R^2^ = 0.9970) and pH 4 (R^2^ = 0.9598), respectively.

The pseudo-second-order adsorption kinetics assumes that the rate of adsorption is predominantly determined by the interaction between the functional groups of the adsorbent surface and the adsorbate dye (step iv). Conversely, the Boyd liquid-film diffusion model evaluates whether the transport of the adsorbate from the liquid phase to the material surface plays a significant role in the adsorption process (step ii). Under neutral conditions, the favorable electrostatic interaction between the anionic carboxyl groups of the CPPCA(1) and the cationic MB dye drives the kinetics of the adsorption process. In contrast, at pH 4, the process is likely limited by the transport of MB from the liquid phase to the CPPCA(1). This limitation can be attributed to the repulsive interactions between MB and H^+^ ions, which may compete for the available binding sites [49], thereby affecting the transport of MB to the surface.

#### 3.2.2. Adsorption Isotherm

**Figure 9:**
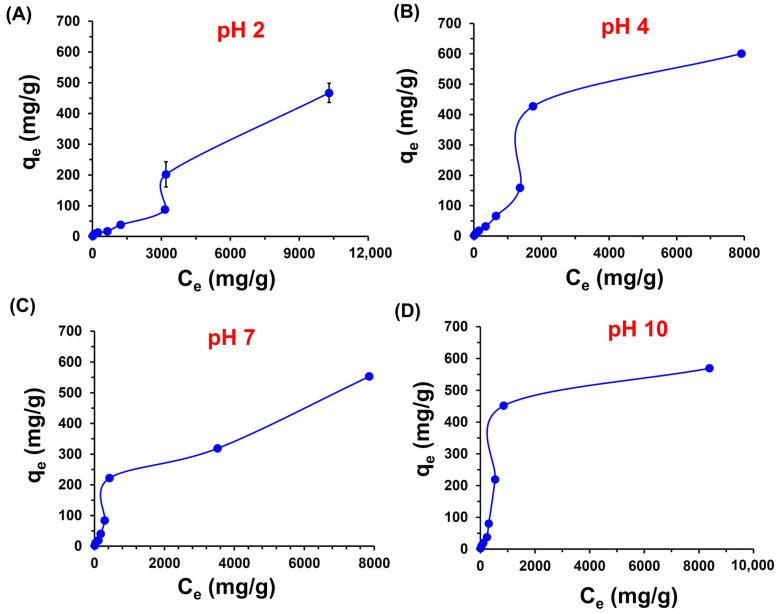
MB adsorption isotherm on CPPCA(1) at 25 °C in various pH media: (**A**) pH 2, (**B**) pH 4, (**C**) pH 7, and (**D**) pH 10. Conditions: temperature = 25 °C, contact time = 24 h, C_o_ = 50 to 20,000 mg/L, and adsorbent dose = 0.2 g/mL. The solid lines serve as a visual guide only.

Figure 9 presents the adsorption isotherm of MB on the CPPCA(1) hydrogel at different pH levels. In acidic conditions (pH 2 and 4), an increase in the equilibrium concentration leads to a rise in q_e_. After reaching a C_e_ of 2000 mg/g, q_e_ increases rapidly, reaching approximately 202 mg/g at pH 2 and 400 mg/g at pH 4. Moreover, at the highest dye concentrations evaluated (20,000 mg/L), the materials achieved peak q_e_ values of 467.1 ± 31.2 mg/g at pH 2 and 600.8 ± 2.1 mg/g at pH 4. In contrast, the isotherms at neutral and basic pH displayed different behaviors, with q_e_ of 522.7 ± 7 mg/g and 569 ± 2.2 mg/g, respectively. Thus, the highest q_e_ values follow the trend: pH 2 < pH 7 < pH 10 < pH 4.

Langmuir, Freundlich, Temkin, and Redlich–Peterson isotherm models were applied to understand the adsorption behavior of MB. Table 2 presents the parameters obtained from the experimental isotherm data along with the R^2^ values for the linear isotherm fitting. The Freundlich model exhibited the best correlation across all tested pH levels. This empirical model describes the adsorption of adsorbate on a heterogeneous surface, accounting for varying energy values associated with the functional groups on that surface [26]. According to the Freundlich model, the adsorbate interacts with the active centers of higher energy first, followed by interactions with lower-energy active centers [50]. This aligns with the characteristics of the CPPCA(1) hydrogel, as the PP is a heterogeneous material primarily composed of lignin, cellulose, and hemicellulose, which are modified by carboxymethyl groups to create active centers with differing energy levels for interaction with the cationic MB. Furthermore, this biohydrogel contains additional functional groups such as hydroxyl, carbonyl, and methyl from the biopolymers present in the PP [51], all of which can interact favorably with the MB dye. Additionally, the chemical crosslinking with CA may promote incomplete reactions, thereby increasing the availability of carboxyl groups for interaction with the dye, as corroborated by the FTIR-ATR results (Figure 4B).

The Freundlich model also assumes that adsorption occurs in multilayers [52], indicating the deposition of MB at higher concentrations. This is particularly evident in the isotherms for pH 2 and 4, where at higher concentrations (C_e_ < 3000 mg/g), the q_e_ increases dramatically, suggesting the formation of multilayers. The Freundlich parameters provide insight into the interactions involved in the adsorption process; specifically, n < 1 indicates that adsorption occurs through chemical interactions, where electrons are shared to form covalent or ionic bonds. Conversely, when n > 1, the adsorption process involves physical adsorption [50]. For the adsorption of MB on the CPPCA(1) hydrogel at different pH levels, the n values ranged from 1.05 to 1.19, indicating that the adsorption of MB across all pH conditions is primarily driven by physical adsorption on the hydrogel surface. Additionally, the 1/n values, which illustrate the adsorption characteristics, were all found to be less than 1, demonstrating that the adsorption process is favorable under all conditions. These favorable interactions are largely attributed to the electrostatic interactions between the cationic MB and the anionic groups of the CPPCA(1) hydrogel, particularly at pH levels above 4, where the carboxyl groups become deprotonated, as indicated by the pH_PZC_ of 3.6. In acidic conditions, the electronegative nitrogen atom in MB may form hydrogen bonding interactions with the –COOH groups of the carboxymethyl substituents and the –OH groups of the polysaccharides present in the PP. Furthermore, hydrophobic interactions and van der Waals forces may also influence MB adsorption. The methyl group of MB is highly hydrophobic and could interact with the alkyl groups present in the PP, particularly in lignin [53,54]. Additionally, at higher concentrations of MB (C_e_ < 3000 mg/g), the formation of multilayers of dye may occur through π–π noncovalent interactions between the π-electrons of the dye’s aromatic rings [55].

The physical adsorption characterized by the Freundlich Isotherm aligns with the thermodynamic parameters found for the MB adsorption process, which is spontaneous, as indicated by the ∆G° values ranging from −4.96 to −2.56 kJ/mol at the tested temperatures (25–50 °C). The ∆H° and ΔS° for the dye adsorption were determined from the linear relationship between ln Kd vs. 1/T (see Figure S8). The results indicate that the adsorption process is both enthalpically (−27.48 kJ/mol) and entropically (77.10 kJ/mol·K) driven. The ∆H° value being less than 40 kJ/mol suggests that the adsorption of MB occurs via physisorption [56]. Furthermore, the positive ΔS° value is likely attributed to the desolvation of water molecules and the desorption of ions from the CPPCA(1) surface, which facilitates MB adsorption. This behavior has also been observed in the adsorption of cationic dyes (such as Crystal Violet and Methyl Red) onto carrageenan hydrogel [57].

**Figure 10:**
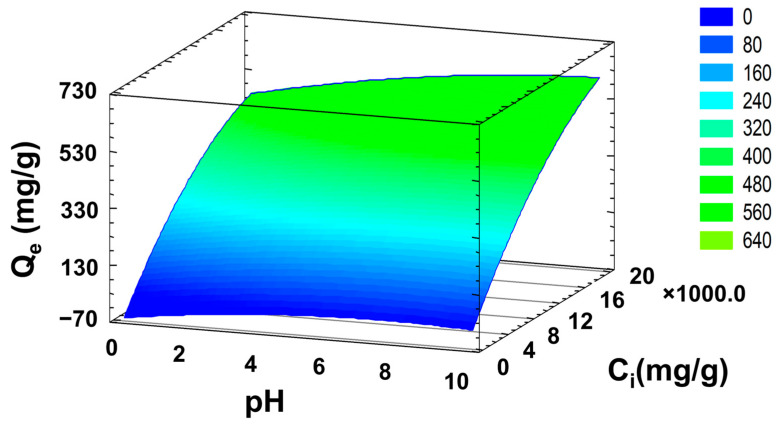
Three-dimensional response surface illustrating the q_e_ as a function of the initial dye concentration (C_o_) and pH in CPPCA(1) hydrogel at 25 °C. Conditions: temperature = 25 °C, contact time = 24 h, C_o_ = 50 to 20,000 mg/L, and adsorbent dose = 0.2 g/mL.

Finally, the experimental data presented in this research demonstrate that pH significantly affects the adsorption profile and capacity of the CPPCA(1) hydrogel, which varies with the C_0_ of the MB exposed to the material. To develop a quadratic model for predicting the q_e_, a surface response of q_e_ as a function of the dye C_i_ and the pH was displayed (Figure 10) using the isothermal experimental data. The surface response shows an R^2^ value of 0.9733, indicating that the model effectively tracks the q_e_ capacity of the CPPCA(1) at different pH values. The process is described by Equation (18).

(18) $$q_{e}=-60.6245+17.9373\left[pH\right]+0.0424262\left[C_{i}\right]-1.23138{\left[pH\right]}^{2}+0.000498686\left[pH\right]\left[C_{i}\right]-8.3412E-7{\left[C_{i}\right]}^{2}$$

Therefore, Equation (18) allows for the prediction of the q_e_ of MB, facilitating the evaluation of the material’s application in removing cationic dyes like MB from contaminated water sources. For MB, the optimization conditions from the quadratic model indicate a concentration of 20,000 mg/L at pH 10; this differs from the maximum q_e_ obtained in this research, which was at pH 4 (600.8 ± 2.1 mg/g). Additionally, it is essential to reevaluate the equation with various cationic dyes to determine its effectiveness in predicting the removal of mono-cationic dyes from water samples. Furthermore, future optimization should be conducted to assess the quadratic model’s ability to track the adsorption of MB dye on a larger scale.

#### 3.2.3. Comparison of CPPCA(1) Hydrogel with Other Adsorbents

Table 3 presents the capacity retention of MB in various materials reported in the literature. The MB retention capacity ranges from 7.5 to 467.5 mg/g for biohydrogels and composites formed with biopolymers. Materials at the micrometric scale show capacities between 86 and 435 mg/g. Both categories exhibit lower retention capacities compared to the PP hydrogel reported in this study. In contrast, when comparing with modified banana peel materials, the CPPCA(1) (q_e_ = 600.8 mg/g) demonstrates approximately two to twenty times the capacity of banana peel modified with Fe_3_O_4_ nanoparticles (q_e_ = 296.4 mg/g) and banana peel modified with Fe (q_e_ = 28.1 mg/g), respectively. This highlights that the chemical modification of PP with carboxymethyl groups presents a promising alternative for creating an efficient material for cationic dye retention. While the formation of nanoparticles to increase surface area and enhance MB retention is an excellent strategy, the production costs of such nanomaterials at a large scale may limit their practical applications.

When comparing CPPCA(1) with other materials derived from polysaccharides, it is evident that the PP hydrogel exhibits lower capacity retention than Starbon^®^ (q_e_ = 845 mg/g), a porous material produced through the pyrolysis of starch. Despite the higher retention capacity of Starbon^®^, its production involves significant energy consumption and requires controlled atmospheric conditions (nitrogen and oxygen) during pyrolysis, limiting its manufacture to specialized laboratories. In contrast, PP is a bioresidue, meaning that recycling and transforming PP into CPPCA(1) hydrogels adds value to the banana production chain. Moreover, commercial chloroacetic acid and CA are economical reagents, with prices ranging from USD 0.55 to USD 0.63 per kilogram without importation from China [70,71]. This affordability facilitates the large-scale production of CPPCA(1) for applications in cationic dye removal from water bodies.

It is also important to highlight that PP is biodegradable and biocompatible, which reduces the negative environmental impact associated with its use compared to the detrimental effects of dyes like MB. Thus, employing the CPPCA(1) hydrogel for dye removal in aquatic environments presents an economical and environmentally friendly alternative. Carboxymethyl materials have proven highly effective for reuse; for instance, carboxymethyl cellulose/carboxylated graphene oxide composites [72] and carboxymethyl cellulose/graphene composite aerogels [73] have been applied in MB adsorption and desorption. These studies demonstrate that materials with carboxymethyl groups can desorb MB dye in an ethanol medium, enabling reuse over up to nine adsorption–regeneration cycles. However, experiments are still needed to evaluate the MB desorption and reusability of the CPPCA(1) hydrogel, ensuring the material can be recovered and reapplied effectively for MB removal from contaminated water.

## 4. Conclusions

In this research, the CPPCA hydrogel was successfully synthesized through sequential reactions, resulting in carboxymethylated PP, which was subsequently crosslinked with CA. ATR-FTIR and TGA analyses confirmed the etherification of PP with carboxymethyl groups and the esterification with CA, demonstrating that the material retains thermal stability up to 160 °C. Additionally, digital photography and SEM images revealed a rough, irregular surface with discontinuous microporosity, confirming the hydrogel formation. The presence of anionic carboxyl groups at pH levels above 4 (pH_PZC_ = 3.6) imparts a negative charge to the hydrogel surface, facilitating electrostatic interactions with cationic MB dye. To evaluate this interaction, CA was utilized at a concentration of 1%, which yielded a high percentage of dye removal (62.2 ± 3.7%) and a gel fraction of 82.8 ± 1.4%. The time-dependent dye adsorption followed the pseudo-second-order and Boyd liquid-film diffusion models at pH 7 and 4, respectively, indicating that the adsorption dynamics are influenced by both electrostatic interactions and the pH of the medium. Furthermore, the Freundlich isotherm model provided the best fit for the adsorption data across different pH levels, highlighting the heterogeneous nature of the adsorption and the formation of multilayers of MB on the hydrogel surface. The CPPCA(1) demonstrated a high capacity retention of 600.8 mg/g at pH 4, indicating its effectiveness in removing cationic dyes. Overall, this economical and environmentally friendly hydrogel shows significant potential for industrial adsorptive processes.

## Figures and Tables

**Table 1:** Kinetic model parameters for MB on CPPCA(1) hydrogel at 25 °C in pH 4 and 7.

Model	Parameter	Units	pH 4	pH 7
Pseudo-first-order	q_e_	mg/g	84.08	30.54
	K_1_	(1/min)	2.76 × 10^−3^	1.61 × 10^−3^
	R^2^		0.8915	0.9248
Pseudo-second-order	q_e_	mg/g	102.04	65.79
	K_2_	g/(mg min)	9.16 × 10^−6^	1.54 × 10^−4^
	R^2^		0.7273	0.9970
Boyd liquid-film diffusion model	K_Fd_	(1/min)	2.50 × 10^−3^	1.50 × 10^−3^
	R^2^		0.9598	0.9348
Elovich	β	g/mg	0.1681	0.0575
	α	mg/(g min)	66.38	0.4300
	R^2^		0.8525	0.8600

**Table 2:** Adsorption isotherm parameters for MB on CPPCA(1) hydrogel at 25 °C.

Isotherm	Parameter	Units	pH 2	pH 4	pH 7	pH 10
Langmuir	q_m_	mg/g	5000.00	1666.67	714.29	833.33
	K_L_	L/mg	8.75 × 10^−6^	7.75 × 10^−5^	3.39 × 10^−4^	2.83 × 10^−4^
	R^2^		0.0060	0.3191	0.8480	0.7458
Freundlich	n		1.16	1.05	1.19	1.05
	1/n		0.86	0.95	0.84	0.95
	K_F_	L/mg	0.11	0.16	0.47	0.26
	R^2^		0.9545	0.9597	0.9209	0.9005
Temkin	B_T_	J mol^−1^	56.48	90.38	81.35	97.82
	K_T_	L/mg	0.010	0.016	0.025	0.023
	R^2^		0.5399	0.6665	0.8225	0.7605
Redlich–Peterson	B	L/mg	0.14	0.05	0.16	0.05
	K_R-P_	L/g	0.114	0.164	0.468	0.265
	R^2^		0.5399	0.6665	0.8225	0.7605

**Table 3:** Comparing CPPCA(1) with various adsorbents to remove MB in water.

Adsorbent	q_e_ (mg/g)	T (°C)	pH	Ref.
Activated carbons from corn cob	333	25	Not reported	[25]
Starbon^®^ produced by pyrolysis of expanded starch	845	35	4	[58]
Residual biomass from *Saccharomyces pastorianus*	204.0	25	3	[59]
Chitosan (biopolymer)/metal–organic frameworks	424.99	25	6	[60]
Starch@Layered material composite films	89.82	25	4.5	[61]
sodium alginate/celite 545 beads	7.5	25	6	[62]
Pectin–alginate–titania microparticles	435	25	Not reported	[63]
Phosphate corn starch nanocrystals	90.79	25	9	[64]
Poly(vinyl alcohol)/chitin/nanocellulose composite	467.5	25	9	[56]
Bentonite–alginate beads	267.14	25	Not reported	[65]
Chitosan/plum kernel shell/TiO_2_ nanoparticles	86.96	25	6	[66]
Alginate/kaolin bead	294.1	25	6	[67]
Fe-modified banana peel	28.1	20	Not reported	[68]
Banana peel with Fe_3_O_4_ nanoparticles	296.4	25	12	[69]
CPPCA(1)	600.8 ± 2.1 *	25	4	This study

* Experimental q_e_ at pH 4.

## Data Availability

The original contributions presented in the study are included in the article/Supplementary Materials, and further inquiries can be directed to the corresponding authors.

## Supplementary Materials

The following supporting information can be downloaded at: https://www.mdpi.com/article/10.3390/polym16223135/s1, Figure S1: Calibration curve of MB in water at 665 nm; Figure S2: Digital photography of CPP after 24 h in distillated water under mechanical stirring at 100 rpm; Figure S3: Elemental composition of CPP and CPPCA(1) according to EDS analysis; Figure S4: ATR-FTIR spectra of CPPCA(x) with different concentrations of CA: (A) 1%, (B) 2%, (C) 3%, (D) 5%, and (E) 10%; Figure S5: Digital photography of CPPCA dry and swollen after 24 h in distillated water. The hydrogels formed with different CA concentrations ranging from 1 to 10%; Figure S6: Digital photography of CPPCA(x) swollen after 24 h in distillated water under mechanical stirring at 100 rpm. The hydrogels formed with different CA concentrations ranging from 1 to 10%; Figure S7: ∆pH versus pH_initial_ curve behavior to determine the point of zero charges (pH_PZC_) in the CPPCA(1); Figure S8: ln Kd vs. 1/temperature for the MB adsorption on CPPCA(1). Conditions: time = 24 h, Co = 2000 mg/L, and adsorbent dose = 0.2 g/mL; Table S1: Thermodynamic parameters for MB adsorption on CPPCA(1) hydrogel.
