# Supplementary material for: Unripe Plantain Peel Biohydrogel for Methylene Blue Removal from Aqueous Solution
Source: Polymers (Basel). 2024 Nov 10;16(22):3135. doi: 10.3390/polym16223135 (PMC11598623; doi:10.3390/polym16223135)
Supplement: Supplementary file 1 [file polymers-16-03135-s001.zip › polymers-3260631-supplementary.pdf]

Supplementary Materials

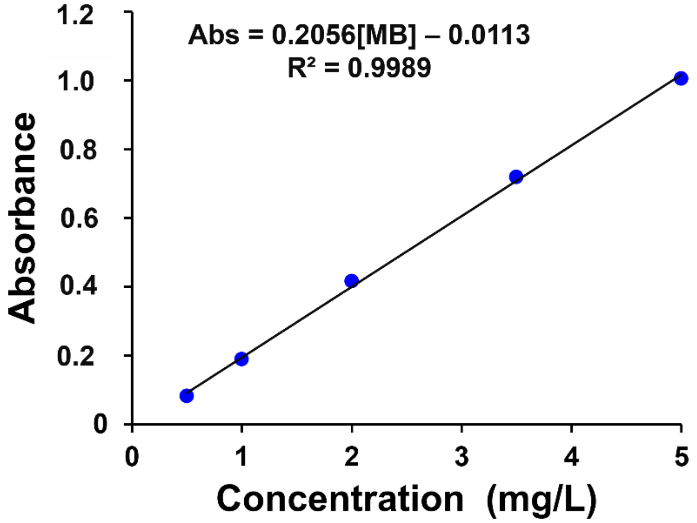

Figure S1. Calibration curve of MB in water at 665 nm.

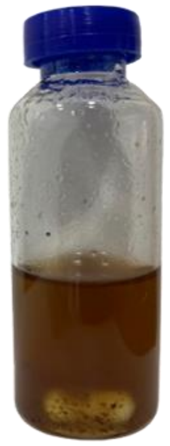

Figure S2. Digital photography of CPP after 24 hours in distillated water under mechanical stirring at 100 rpm.

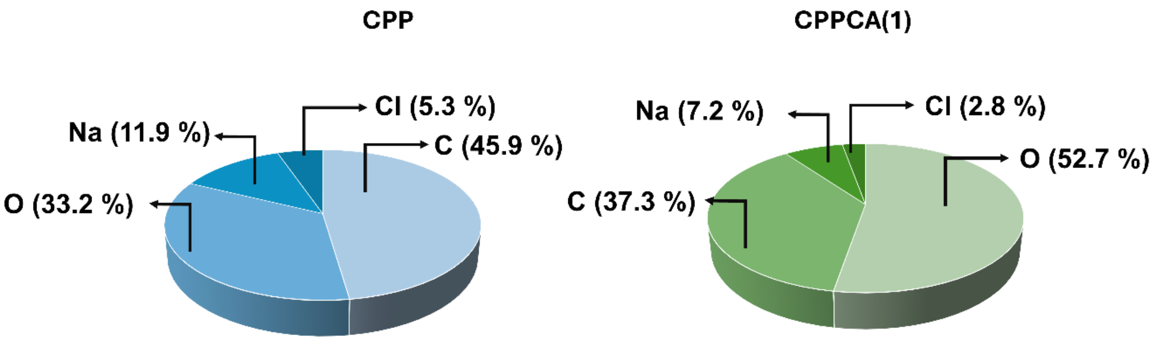

Figure S3. Elemental composition of CPP and CPPCA(1) according to EDS analysis.

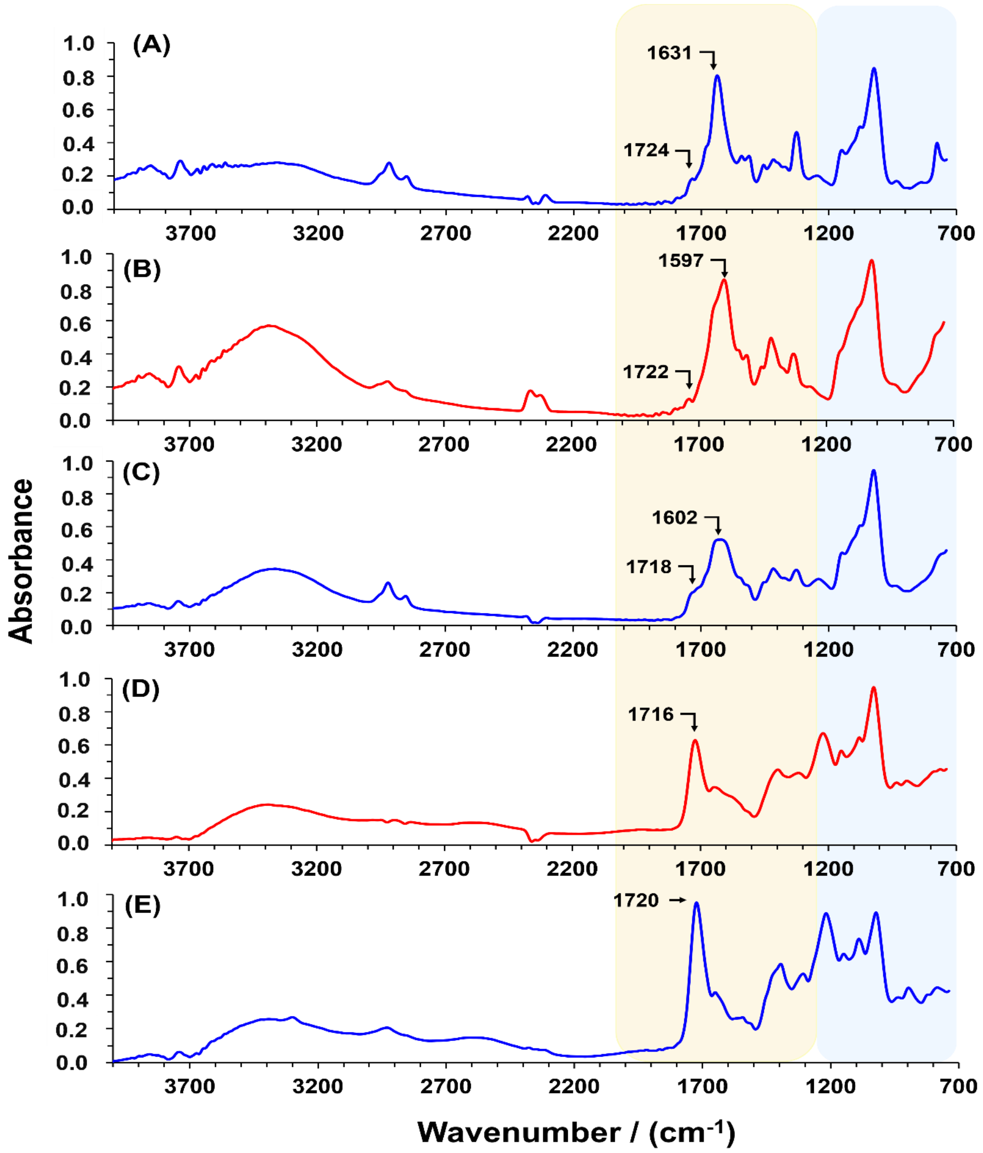

Figure S4. ATR-FTIR spectra of CPPCA(x) with diferent concentration of CA: (A) 1 %, (B) 2 %, (C) 3 %, (D) 5 %, and (E) 10 %.

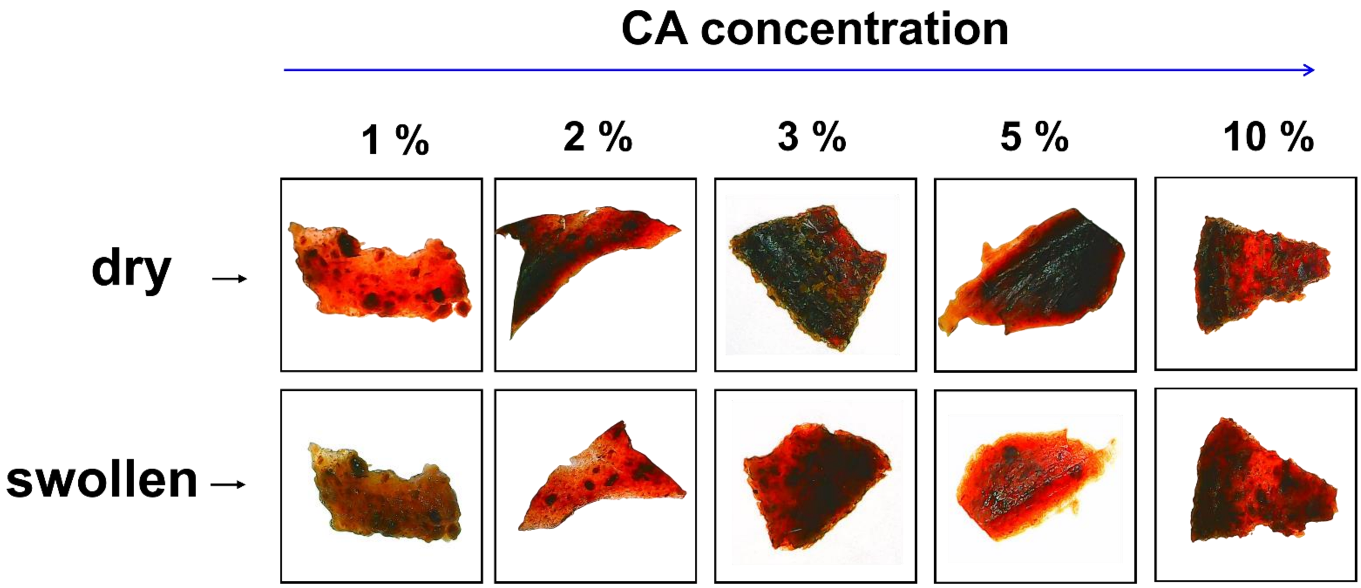

Figure S5. Digital photography of CPPCA dry and swollen after 24 hours in distillated water. The hydrogels formed with different CA concentration ranging from 1 to 10 %.

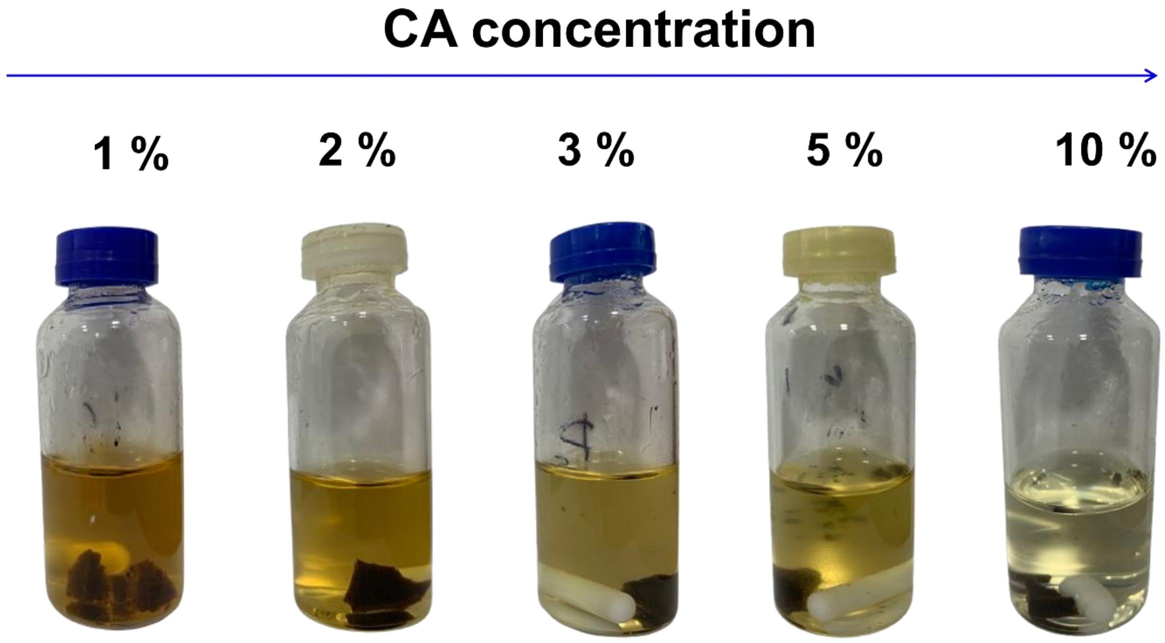

Figure S6. Digital photography of CPPCA(x) swollen after 24 hours in distilled water under mechanical stirring at 100 rpm. The hydrogels formed with different CA concentration ranging from 1 to 10 %.

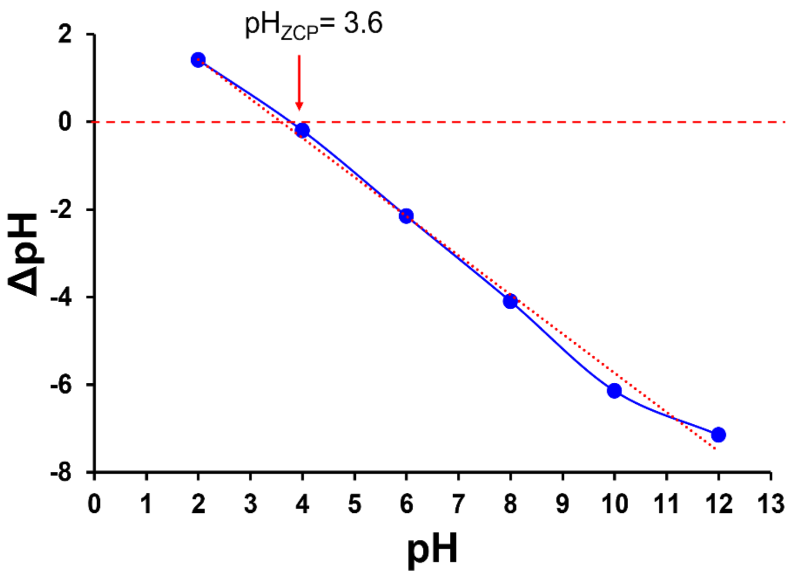

Figure S7. ΔpH versus $pH_{initial}$ curve behavior to determinate of the point of zero charge (pH_{PZC}) of the CPPCA(1).

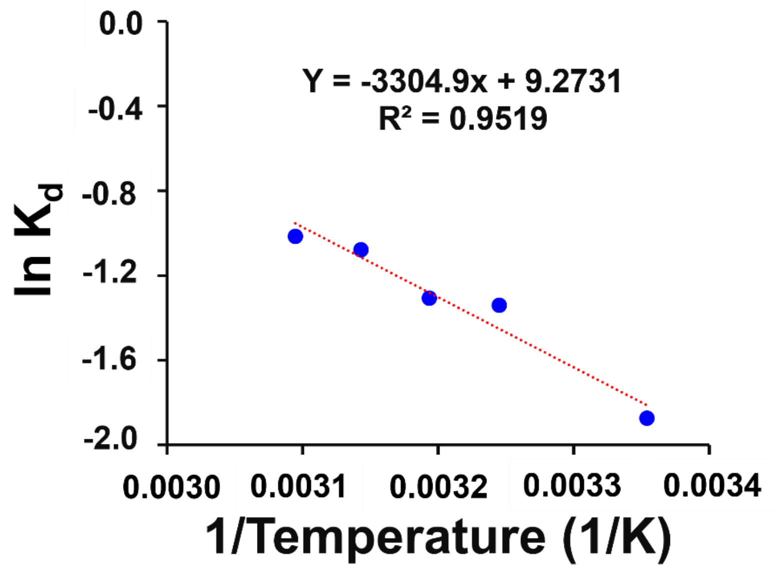

Figure S8. ln K_d vs 1/temperature for the MB adsorption on CPPCA(1). Conditions: time = 24 h, C_o= 2000 mg/L, and adsorbent dose = 0.2 g/mL.

Table S1. Thermodynamic parameters for MB adsorption on CPPCA(1) hydrogel

Temperature (°C)	ΔG° (kJ/mol)	ΔS° (J/mol K)	ΔH° (J/mol K)
25	-4.96		
35	-3.49		
40	-3.51	77.10	-27.48
45	-2.67		
50	-2.56		
